# Tolerance Modelling of Vibrations and Stability for Periodic Slender Visco-Elastic Beams on a Foundation with Damping. Revisiting

**DOI:** 10.3390/ma13183939

**Published:** 2020-09-06

**Authors:** Jarosław Jędrysiak

**Affiliations:** Department of Structural Mechanics, Łódź University of Technology, al. Politechniki 6, 90-924 Łódź, Poland; jarek@p.lodz.pl; Tel.: +48-42-631-3564

**Keywords:** periodic beam, microstructure, tolerance modelling, effect of microstructure

## Abstract

The mathematical modelling of certain problems of vibrations and stability for periodic slender visco-elastic beams is presented in this note. To consider these problems and take into account the effect of the microstructure, the tolerance modelling approach is proposed. Using this technique, the equation with non-continuous, periodic, highly oscillating coefficients is replaced by a system of differential equations with constant coefficients. Moreover, these governing equations describe the effect of the microstructure on the overall behavior of the beams under consideration. The tolerance modelling can lead to equations of two different tolerance models—the standard and the general, under weakened assumptions. This averaging tolerance method was assessed by comparison with the asymptotic homogenization, the governing equations of which omit this effect. My considerations were limited to proposing and presenting only mathematical models describing investigated beams. In a simple analytical example, the application of the presented average models is shown.

## 1. Introduction

**Figure 1:**
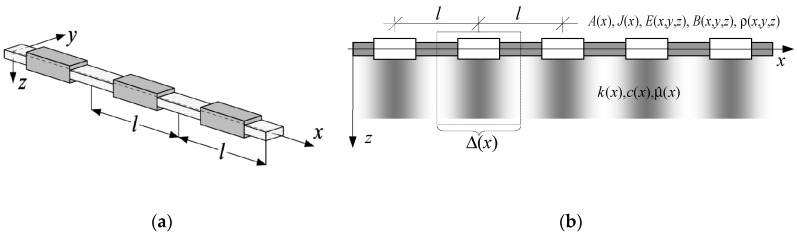
Fragments of periodic beams: (**a**) without foundation; (**b**) on a foundation.

Problems concerning the vibrations and stability of periodic, slender, visco-elastic beams were investigated in this contribution. Such beams can interact with a heterogeneous damping foundation. These beams have a periodic structure along their axis *x* and are made of many identical small repeatable elements, called periodic cells; cf. Figure 1 with fragments of the beams.

Although beams are one of the simplest examples of periodical structures, they are often and widely used in different branches of engineering. Moreover, periodic objects can represent approximate models of some more complex systems. 

The dynamics and stability of periodic, slender visco-elastic beams on a periodic damping foundation are described by partial differential equations with non-continuous, periodic, highly oscillating coefficients. The analysis of those problems using the aforementioned equations is rather difficult. Hence, various simplified models of such beams were proposed. Some of these models use the concept of the effective (averaged) stiffness (and other properties) of the beams. Among them, one can mention the models which are based on the theory of asymptotic homogenization (cf. Bensoussan et al. [1]), used for instance to analyze periodic plates; cf. Kohn and Vogelius [2]. This theory was used to consider equilibrium equations of microperiodic beams by Kolpakov [3,4,5]. Various other modelling methods were also proposed to investigate different composite media; e.g., Matysiak and Nagórko [6] showed a homogenization with microlocal parameters for periodic plates; a comprehensive study on composite beams vibrations was done by Hajianmaleki and Qatu [7]; Roque et al. [8] formulated a certain model of bending for laminated composite beams, capturing the effects of the microstructure, using a modified couple stress theory and a meshless method; Batra and Xiao [9] proposed a layer-wise third order shear and normal deformable plate/shell theory incorporating all geometric nonlinearities to study finite deformations of curved laminated beams; Grygorowicz et al. [10], Wittenbeck et al. [11] and Grygorowicz and Magnucka-Blandzi [12] analyzed dynamic stability of sandwich beams having variable mechanical properties of the core; a torsion of composite beams with a phase made of auxetic material was investigated by Stręk et al. [13] and Jopek and Stręk [14] using an analytical-numerical model based on analytical relations and finite element method; Pawlus [15] modelled the stability of three-layered annular plates made of fiber-reinforced composite facings and a foam core; some numerical investigations using a new approximation function in Carrera’s unified formulation for composite layered beams were shown by Arruda et al. [16].

Other theoretical and numerical results were shown in many papers for composite functionally-graded structures. Bui et al. [17] applied meshless methods to analyze natural frequencies of sandwich beams with functionally graded composite core. Murin et al. [18] considered the effect of the shear correction function in modal analysis of functionally graded composite beams. A new theory with a generalization of layer-wise displacement approaches to dynamics of composite curved beams was proposed by Carpentieri et al. [19]. A free vibration optimization of composite functionally graded nano-beams was analyzed by Roque et al. [20]. Fantuzzi and Tornabene [21] proposed a strong formulation isogeometric analysis for laminated composite plates. A layer-wise theory and a differential quadrature method for composite plates were shown by Liu et al. [22]. Awrejcewicz et al. [23] presented a mathematical model of composite beams using the couple stress theory and size-dependent governing equations of the layers’ motions. A semi-analytical method for stability problems of columns with closed cross-sections made of composite fiber-multilayered plate was proposed by Kołakowski and Mania [24] and Mania et al. [25] using the classical laminate plate theory. Eringen’s nonlocal theory was applied to investigate the bending of composite functionally graded beams with internal porosity by Jouneghani et al. [26]. Ghayesh [27] and Ghayesh and Farokhi [28] proposed a vibration analysis of axially functionally-graded composite beams.

However, the effect of the microstructure size (cf. Brillouin [29]) is usually omitted in governing equations of these models. Hence, these models cannot be good tools to investigate this effect. However, this effect can play a role in the different behaviors of microheterogeneous structures; cf. [29,30]. That was observed and analyzed in a few papers; e.g., the differential quadrature method was applied to analyze vibration band gaps in periodic beams by Xiang and Shi [31] and to consider flexural wave band gaps in periodic composite plates by Cheng et al. [32]; the transfer matrix method was adapted to analysis of flexural wave propagation in a beam on an elastic foundation and for investigating natural frequencies of non-uniform periodic beams by Yu et al. [33] and Xu et al. [34], respectively; wave propagation in beams with periodically varying stiffness was considered by Chen [35] via applying the multireflection method; Zhi-Jing et al. [36] investigated vibration band gap properties of periodic Mindlin’s plates using a spectral element method; a center finite difference method was used by Zhou et al. [37,38] to analyze band gaps of periodic thin plates with or without damping. 

However, in governing equations of these models for various composite structures, the size of the microstructure parameter (the size of the periodicity cell) is often neglected. Therefore, non-asymptotic averaged models of the periodic composite structures are introduced, which are usually called the tolerance models. They are derived by applying the tolerance modelling technique; cf. Woźniak and Wierzbicki [39], Woźniak et al. [40] and Woźniak et al. [41]. 

The obtained tolerance model equations have constant coefficients, and in contrast to other averaged models, some of them depend on the size of the microstructure. The proposed method can be adopted to any differential equations with highly oscillating periodic or tolerance-periodic coefficients. This approach, in contrast to the asymptotic homogenization, allows one to analyze the effect of the microstructure size. 

Various dynamical, stability and thermoelastic problems of periodic structures and composites were considered using this method. These applications can be found in a series of papers; e.g., the dynamics of microperiodic beams were analyzed by Mazur-Śniady [42]; periodic fluid-saturated grounds were considered by Dell’Isola et al. [43]; Wierzbicki and Woźniak [44] investigated the dynamics of plane periodic structures; Jędrysiak [45] studied the stability problems of periodic plates; an analysis of vibrations for periodic wavy-type plates was shown by Michalak [46]; Nagórko and Woźniak [47] considered the dynamics of thin plates reinforced periodically by stiffeners; Baron [48] analyzed vibrations of periodic medium-thickness plates; buckling and free vibrations of periodic thin plates on a foundation were investigated by Jędrysiak [49,50]; an application to dynamics of periodic thin plates with the microstructure size of an order of the plate thickness was presented by Mazur-Śniady et al. [51]; Tomczyk [52,53] analyzed the stability of periodic shells; the nonlinear dynamics of visco-elastic periodic plates were modelled by Jędrysiak [54]; Domagalski and Jędrysiak [55] studied the geometrically nonlinear vibrations of periodic beams; vibrations of periodic three-layered plates were shown by Marczak and Jędrysiak [56]; free vibrations of periodic thin plates with uncertain material properties were investigated by Jędrysiak and Ostrowski [57].

Different thermomechanical problems of functionally graded media with microstructures were also considered using the tolerance modelling approach; e.g., Michalak and Wirowski [58], Perliński et al. [59] and Wirowski et al. [60] analyzed dynamics and stability of plates with longitudinally graded structures with or without an elastic foundations; free vibrations of transversally graded thin plates on an elastic foundation were presented by Jędrysiak and Kaźmierczak-Sobińska [61]; the dynamics of a thin-walled structure with a dense system of ribs was considered by Michalak [62]; Ostrowski and Michalak [63] showed an application for heat conduction in cylindrical composite conductors with non-uniform microstructure; the effect of microstructure in thermoelastic problems of transversally graded laminates was investigated by Pazera and Jędrysiak [64]; vibrations of thin plates with the microstructure size of an order of the plate thickness were analyzed by Jędrysiak [65]; Tomczyk and Szczerba [66] proposed a new asymptotic-tolerance model of dynamics for thin microstructured transversally graded shells; Jędrysiak [67] considered free vibrations of medium thickness functionally graded plates with a microstructure. 

In this work, two new tolerance models of slender periodic beams are proposed—one (which can be called the “standard” or “classic” tolerance model) under the concept of slowly-varying functions and the second (it can be named “*general tolerance model*”) under the concept of weakly-slowly-varying functions—suggested and shown also for thin periodic plates by Jędrysiak [68]. Governing equations of both the models describe the effect of the microstructure on the overall behavior of the periodic beams under consideration. The microstructural effect of periodic beams on a foundation plays a crucial role, for instance, in dynamical problems such as free vibrations or forced vibrations. Using the tolerance models one can investigate the higher order frequencies (and higher order vibrations; cf. Brillouin [29]) related to the microstructure of the beam, not only fundamental lower frequencies (and vibrations) related to the macrostructure of the beam. Moreover, these model equations are assessed by comparison with the governing equations obtained using the asymptotic homogenization, which omits this effect. Considerations in this note are restricted only to a theoretical analysis and derivation of the problem. An application of the above-mentioned models is shown in a simple example for an infinite periodic beam.

## 2. Modelling Approach

### 2.1. Modelling Preliminaries

Denote by *Oxyz* the orthogonal Cartesian coordinate system and by *t* the time coordinate. Let the region of the undeformed beam be denoted by Ω≡{(x,y,z):−a/2≤y≤a/2, −h/2≤z≤h/2, x∈Λ}, where Λ is the beam axis; Λ ≡ [0,*L*]; *h*(⋅) is the beam height; *a*(⋅) is the beam width; *L* is the length of the beam. Derivatives of *x* are denoted by ∂. The “basic cell” on *Ox* is defined as Δ ≡ [−*l*/2,*l*/2]. Hence, parameter *l* is the length of cell Δ. It is assumed that this parameter satisfies condition *h*_max_ <<*l*<< *L* and is called the microstructure parameter. Geometrical properties of the beam—height *h*(·) and width *a*(·); and foundation properties—Winkler’s coefficient *k*(·), damping parameter *c*(·) and mass density of foundation $\hat{\mu }(\cdot )$ can be periodic functions in *x*, but material properties of the beam—modulus of elasticity *E* = *E*(·,*y*,*z*), modulus of visco-elasticity *B* = *B*(·,*y*,*z*) and mass density ρ = ρ(·,*y*,*z*) can be periodic functions in *x* and even functions in *y*, *z*. Let *u*(*x*,*z*,*t*) ($x\in \bar{\Delta }$) be a vertical beam displacement (along *z*-axis) and *q* be total loadings in the *z*-axis direction.

In the modelling the assumptions and relations of the slender beam theory are applied:

● *The kinematic assumption of slender beams.*

(1) u(x,z,t)=−z∂w(x,t), w(x,z,t)=w(x,t),

where *w*(·,*t*) is the beam deflection.

The similar form to Equation (1) is introduced for virtual displacements:

(2) $$\bar{u}(x,z)=-z\partial \bar{w}(x),\;\bar{w}(x,z)=\bar{w}(x),$$

where: $\bar{u}(\cdot )$—the virtual beam displacement; $\bar{w}(\cdot )$—the virtual beam deflection.

● *The strain–displacement relation.*

(3) e=∂u,

where: *e*—the beam strain.

● *The stress–strain relation.*

(4) $$s=s_{0}+Ee+B\dot{e},$$

where: *s*—the stress; *s*_0_—the initial stress; *E*(·), *B*(·)—modulus of: elasticity, visco-elasticity, respectively (being periodic functions in *x*).

● *The virtual work equation*

(5) $$\int_{\Lambda }\int_{-a/2}^{a/2}\int_{-h/2}^{h/2}\rho \ddot{u}\bar{u}dzdydx+\int_{\Lambda }\int_{-a/2}^{a/2}\int_{-h/2}^{h/2}\rho \ddot{w}\bar{w}dzdydx+\int_{\Lambda }\int_{-a/2}^{a/2}\int_{-h/2}^{h/2}s\bar{e}dzdydx=\int_{\Lambda }q\bar{w}dx-\int_{\Lambda }(kw+\hat{\mu }\ddot{w}+c\dot{w})\bar{w}dx,$$

where: ρ(·)—the mass density of the beam material; *k*(·)—the Winkler’s coefficient; *E*, *B*—modulus of: elasticity, visco-elasticity, respectively (being periodic functions in *x*); $\bar{e}(\cdot )$—the virtual beam strain.

Denoting stiffnesses of the plate: bending *d*(·), visco-elastic *b*(·); the mass density μ(·); the rotational mass inertia *j*(·), being periodic functions in *x*, in the form:

(6) $$\begin{array}{ll} d(x)=\int_{-a/2}^{a/2}\int_{-h/2}^{h/2}E(x,y,z)z^{2}dzdy, & b(x)=\int_{-a/2}^{a/2}\int_{-h/2}^{h/2}B(x,y,z)z^{2}dzdy,\\ \mu (x)=\int_{-a/2}^{b/2}\int_{-h/2}^{h/2}\rho (x,y,z)dzdy, & j(x)=\int_{-a/2}^{a/2}\int_{-h/2}^{h/2}\rho (x,y,z)z^{2}dzdy, \end{array}$$

and the axis force by:

(7) $$n=\int_{-a/2}^{a/2}\int_{-h/2}^{h/2}s_{0}dzdy,$$

after some manipulations of Equation (5) combined with Equations (1)–(4), by applying the divergence theorem and the du Bois–Reymond lemma, the following governing equations can be derived:

● *The constitutive equation*

(8) $$m=d\partial \partial w+b\partial \partial \dot{w},$$

● *The equation of motion*

(9) $$\partial \partial m-\partial (n\partial w)+\mu \ddot{w}-j\partial \partial \ddot{w}+kw+\hat{\mu }\ddot{w}+c\dot{w}=q.$$

Substituting Equation (8) into Equation (9), the governing equations of the periodic beams can be written as:

(10) $$\partial \partial (d\partial \partial w)+\partial \partial (b\partial \partial \dot{w})-\partial (n\partial w)+\mu \ddot{w}-j\partial \partial \ddot{w}+kw+\hat{\mu }\ddot{w}+c\dot{w}=q.$$

The above equation, similarly to Equations (8)–(9), has highly oscillating, non-continuous, periodic functional coefficients.

### 2.2. Introductory Concepts of the Tolerance Modelling

Concepts defined in books [1,2,3] are applied in modelling. They are also presented various papers, e.g., in [67], but some of them are reviewed below in the form adopted for beams.

Let Δ(*x*) ≡ *x* + Δ be a cell at *x* ∈ Λ_Δ_, Λ_Δ_ = {*x* ∈ Λ: Δ(*x*) ⊂ Λ}. The averaging operator for an integrable function *f* is defined by

(11) $$<f>(x)=l^{-1}\int_{\Delta (x)}f(\xi )d\xi ,\;x\in \Lambda ,\;\xi \in \Delta (x).$$

For function *f*, a periodic one in *x*, the averaged value calculated from (11) is constant.

Let $\partial^{k}f$ denote the *k*–th gradient of function f=f(x), x∈Λ, k=0,1,…,α, (α≥0 and for considered problems of beams α = 2); $\partial^{0}f\equiv f$; and ${\tilde{f}}^{\left(k\right)}(\cdot ,\cdot )$ be a function defined in $\bar{\Lambda }\times R^{m}$.

Function $f\in H^{2}(\Lambda )$ is the tolerance-periodic function, $f\in TP_{\delta }^{2}(\Lambda ,\Delta )$, if for k=0,1,…,2, the following conditions are satisfied:

- $\left(\forall x\in \Lambda )\;(\exists {\tilde{f}}^{\left(k\right)}(x,\cdot )\in H^{0}(\Delta )\right)$$\left[||\partial^{k}f|{}_{\Lambda_{\Delta }}(\cdot )-{\tilde{f}}^{\left(k\right)}(x,\cdot )|{|}_{H^{0}(\Lambda ,\Delta )}\leq \delta \right]$;
- $\int_{\Delta (\cdot )}{\tilde{f}}^{\left(k\right)}(\cdot ,\xi )d\xi \in C^{0}(\bar{\Lambda })$.

where function ${\tilde{f}}^{\left(k\right)}(x,\cdot )$ is the periodic approximation *of* $\partial^{k}f$ *in* Δ(x), x∈Λ, k=0,1,…,2; and δ is the tolerance parameter, δ << 1, related to the considered problems.

Function $F\in H^{2}(\Lambda )$ is the weakly-slowly-varying function, $F\in WSV_{\delta }^{2}(\Lambda ,\Delta )$, if

- $F\in TP_{\delta }^{2}(\Lambda ,\Delta )$;
- (∀(x;ξ)∈Λ) [(x;ξ)⇒F(x)≈F(ξ)∧∂F(x)≈∂F(ξ)].

Function $F\in H^{2}(\Lambda )$ is *the slowly-varying function*, $F\in SV_{\delta }^{2}(\Lambda ,\Delta )$, if

- $F\in WSW_{\delta }^{2}(\Lambda ,\Delta )$;
- $(\forall x\in \Lambda )\;l|\partial F(x)|{|}_{\Delta (x)}\approx 0$.

Function $\varphi \in H^{2}(\Lambda )$ is *the highly oscillating function*, $\varphi \in HO_{\delta }^{2}(\Lambda ,\Delta )$, if

- $\varphi \in TP_{\delta }^{2}(\Lambda ,\Delta )$;
- $\left(\forall x\in \Lambda )\;[{\tilde{\varphi }}^{\left(k\right)}(x,\cdot ){|}_{\Delta (x)}=\partial^{k}\tilde{\varphi }(x),\;k=0,1,\ldots ,2\right]$.
- $\forall F\in SV_{\delta }^{2}(\Lambda ,\Delta )$$\exists f\equiv \varphi F\in TP_{\delta }^{2}(\Lambda ,\Delta )$ ${\tilde{f}}^{\left(k\right)}(x,\cdot ){|}_{{}_{\Delta (x)}}=F(x)\partial^{k}\tilde{\varphi }(x){|}_{{}_{\Delta (x)}},\;k=1,2$.

For *k* = 0 let us denote $\tilde{f}\equiv {\tilde{f}}^{\left(0\right)}$.

Let *g*(⋅) be a highly oscillating function, defined on $\bar{\Lambda }$, $g\in HO_{\delta }^{2}(\Lambda ,\Delta )$, continuous together with gradient ∂^1^*g*. Gradient ∂^2^*g* is a piecewise continuous and bounded. Function *g*(⋅) is the fluctuation shape function of the second kind, $FS_{\delta }^{2}(\Lambda ,\Delta )$, if it depends on *l* as a parameter and the following conditions hold:

- ∂*^k^g* ∈ *O*(*l*^α−*k*^) for *k* = 0,1, …,α, α = 2, ∂^0^*g* ≡ *g*;
- <*g*>(*x*) ≈ 0 for every $x\in \Lambda_{\Delta }$,

with the microstructure parameter *l*. The above second condition can be replaced by <μ*g*>(*x*) ≈ 0 for every *x* ∈ Λ_Δ_, where μ > 0 is a certain periodic function.

### 2.3. Tolerance Modelling Assumptions

The introductory concepts are applied in the formulation of the modelling assumptions; cf. [1,2,3].

The first assumption is the micro-macro decomposition, introduced in the form:

(12) $$w(x,t)=W(x,t)+g^{A}(x)V^{A}(x,t),\;A=1,\ldots ,P,\;x\in \Lambda ,$$

where $W(\cdot ,t),\;V^{A}(\cdot ,t)\in WSV_{\delta }^{2}(\Lambda ,\Delta )$ or $W(\cdot ,t),\;V^{A}(\cdot ,t)\in SV_{\delta }^{2}(\Lambda ,\Delta )$; i.e., they are weakly-slowly-varying functions of the second kind or slowly-varying functions of the second kind, respectively. Functions *W*(⋅,*t*) and *V^A^*(⋅,*t*) are new kinematic unknowns, named the macrodeflection and the fluctuation amplitudes, respectively. Moreover, *g^A^*(⋅), *A*, *B* = 1,…,*P*, are the known fluctuation shape functions, which are usually postulated a priori in the considered problem and describe the unknown field (here: the deflection) oscillations caused by the beam inhomogeneity. These functions, apart from the periodicity condition, have to satisfy the following restrictions:

- ∂*^k^g^A^*∈*O*(*l*^2^^−*k*^) for *k* = 0, 1, …, 2;
- <μ*g^A^*> = 0;
- <μ*g^A^g^B^*> = 0 for *A* ≠ *B*, *A*, *B* = 1, …,*P*.

The second assumption is the tolerance averaging approximation, in which it is assumed that terms *O*(δ) are negligibly small, e.g., for $f\in TP_{\delta }^{2}(\Lambda ,\Delta ),$ $F\in SV_{\delta }^{2}(\Lambda ,\Delta )$ or $F\in WSV_{\delta }^{2}(\Lambda ,\Delta )$ $g^{A}\in FS_{\delta }^{2}(\Lambda ,\Delta ),$ in:

(13) $$\begin{array}{l} <f>(x)=<\bar{f}>(x)+O(\delta ),\\ <fF>(x)=<f>(x)F(x)+O(\delta ),\\ <f\partial (g^{A}F)>(x)=<f\partial g^{A}>(x)F(x)+O(\delta ). \end{array}$$

The third assumption is the axis force restriction, in which it is assumed that terms involving fluctuating parts of the axis force can be neglected in comparing to terms with averaged parts; i.e.,:

(14) $$\begin{array}{l} n(x)=N(x)+\tilde{n}(x),\\ N=<n>,\;\;\;\;\;<\tilde{n}>=0, \end{array}$$

where $N(\cdot )\in SV_{\delta }^{2}(\Lambda ,\Delta )$ and $\tilde{n}(\cdot )\in TP_{\delta }^{2}(\Lambda ,\Delta )$ are averaged and fluctuating parts of axis force, respectively.

### 2.4. The Outline of the Tolerance Modelling Procedure

The modelling procedure can be outlined following [1], [2] or [3], but here it is similar to that modified one shown in [1]. The starting point of the modelling is the formulation of the virtual work equation in the form Equation (5). From the combining Equation (5) with Equations (1)–(4), using the divergence theorem and the du Bois–Reymond lemma, after some manipulations the governing equations of the beam can be derived in the form of Equations (8)–(9) or (10).

Now, the first step of the tolerance modelling procedure is the substitution of the micro-macro decomposition (12) into Equation (10). In this case the governing dynamic Equation (10) does not hold; i.e., in the framework of macrodynamics there exists a residual field *r*(·) defined by:

(15) $$\begin{array}{l} r=\partial \partial (d\partial \partial (W+g^{A}V^{A})+b\partial \partial (\dot{W}+g^{A}{\dot{V}}^{A}))-\partial (n\partial (W+g^{A}V^{A}))+\\ \;\;\;+\mu (\ddot{W}+g^{A}{\ddot{V}}^{A})-j\partial \partial (\ddot{W}+g^{A}{\ddot{V}}^{A})+\\ \;\;\;+k(W+g^{A}V^{A})+\hat{\mu }(\ddot{W}+g^{A}{\ddot{V}}^{A})+c(\dot{W}+g^{A}{\dot{V}}^{A})-q. \end{array}$$

The next assumption is *t*he residual orthogonality condition, in which the residual field *r*(·) has to satisfy the following conditions:

(16) $$<r>(x,t)=0,\;\;<rg^{B}>(x,t)=0.$$

The above conditions (16), together with the assumptions—the tolerance averaging approximation (13) and the axis force restriction (14)—lead to a system of equations for the macrodeflection *W*(·,*t*) and the fluctuation amplitudes: *V^A^*(·,*t*), *A* = 1,...,*P*. The form of these governing equations depends on the specification of the class of slowly-varying functions *W*(·,*t*), *V^A^*(·,*t*) (weakly-slowly-varying or slowly-varying function).

## 3. Results—The Governing Equations

### 3.1. The General Tolerance Model Equations

By using the residual orthogonality condition Equation (16) with the modelling assumptions (12)–(14), introducing following denotations of averaged coefficients:

(17) $$\begin{array}{lll} D\equiv <d>, & D^{A}\equiv <d\partial \partial g^{A}>, & D^{AB}\equiv <d\partial \partial g^{A}\partial \partial g^{B}>,\\ \tilde{B}\equiv <b>, & {\tilde{B}}^{A}\equiv <b\partial \partial g^{A}>, & {\tilde{B}}^{AB}\equiv <b\partial \partial g^{A}\partial \partial g^{B}>,\\ \tilde{m}\equiv <\mu >, & {\tilde{m}}^{A}\equiv l^{-2}<\mu g^{A}>, & {\tilde{m}}^{AB}\equiv l^{-4}<\mu g^{A}g^{B}>,\\ \vartheta \equiv <j>, & \vartheta^{A}\equiv l^{-1}<j\partial g^{A}>, & \vartheta^{AB}\equiv l^{-2}<j\partial g^{A}\partial g^{B}>,\\ C\equiv <c>, & C^{A}\equiv l^{-2}<cg^{A}>, & C^{AB}\equiv l^{-4}<cg^{A}g^{B}>,\\ K\equiv <k>, & K^{A}\equiv l^{-2}<kg^{A}>, & K^{AB}\equiv l^{-4}<kg^{A}g^{B}>,\\ \hat{m}\equiv <\hat{\mu }>, & {\hat{m}}^{A}\equiv l^{-2}<\hat{\mu }g^{A}>, & {\hat{m}}^{AB}\equiv l^{-4}<\hat{\mu }g^{A}g^{B}>,\\ N\equiv <n>, & N^{AB}\equiv l^{-2}<n\partial \partial g^{A}g^{B}>, & {\overset{⌣}{N}}^{AB}\equiv -l^{-2}<n\partial g^{A}\partial g^{B}>=-N^{AB},\\ Q\equiv <q>, & Q^{A}\equiv l^{-2}<qg^{A}>; & \\ {\bar{N}}^{A}\equiv l^{-2}<ng^{A}>, & {\bar{N}}^{AB}\equiv l^{-4}<ng^{A}g^{B}>, & \\ {\bar{D}}^{A}\equiv l^{-2}<dg^{A}>, & {\bar{D}}^{AB}\equiv l^{-4}<dg^{A}g^{B}>, & \\ {\hat{D}}^{AB}\equiv l^{-2}<d\partial g^{A}\partial g^{B}>, & {\overset{⌣}{D}}^{AB}\equiv l^{-2}<d\partial \partial g^{A}g^{B}>, & \\ {\bar{B}}^{A}\equiv l^{-2}<bg^{A}>, & {\bar{B}}^{AB}\equiv l^{-4}<bg^{A}g^{B}>, & \\ {\hat{B}}^{AB}\equiv l^{-2}<b\partial g^{A}\partial g^{B}>, & {\overset{⌣}{B}}^{AB}\equiv l^{-2}<b\partial \partial g^{A}g^{B}>, & \\ {\bar{\vartheta }}^{A}\equiv l^{-2}<jg^{A}>, & {\bar{\vartheta }}^{AB}\equiv l^{-4}<jg^{A}g^{B}>, & {\tilde{\vartheta }}^{AB}\equiv l^{-3}<jg^{A}\partial g^{B}>; \end{array}$$

the equations for *W*(⋅,*t*) and *V^A^*(⋅,*t*) are derived:

- The general tolerance constitutive equations.
  (18) $$\begin{array}{l} M=D\partial \partial W+D^{A}V^{A}+\underline{l^{2}{\bar{D}}^{A}\partial \partial V^{A}}+\tilde{B}\partial \partial \dot{W}+{\tilde{B}}^{A}{\dot{V}}^{A}+\underline{l^{2}{\bar{B}}^{A}\partial \partial {\dot{V}}^{A}},\\ M^{A}=D^{A}\partial \partial W+D^{AB}V^{B}+\underline{l^{2}{\overset{⌣}{D}}^{AB}\partial \partial V^{B}}+{\tilde{B}}^{A}\partial \partial \dot{W}+{\tilde{B}}^{AB}{\dot{V}}^{B}+\underline{l^{2}{\overset{⌣}{B}}^{AB}\partial \partial {\dot{V}}^{B}}; \end{array}$$
- The general tolerance equations of motion.
  (19) $$\begin{array}{l} \partial \partial M-\partial (N\partial W)+(\tilde{m}+\hat{m})\ddot{W}+l^{2}({\tilde{m}}^{A}+{\hat{m}}^{A}){\ddot{V}}^{A}-\partial (\vartheta \partial \ddot{W})-l\partial (\vartheta^{A}{\ddot{V}}^{A})+\\ +KW+l^{2}K^{A}V^{A}+C\dot{W}+l^{2}C^{A}{\dot{V}}^{A}-\underline{l^{2}\partial ({\bar{N}}^{A}\partial V^{A}+{\bar{\vartheta }}^{A}\partial {\ddot{V}}^{A})}=Q,\\ M^{A}+l^{2}({\tilde{m}}^{A}+{\hat{m}}^{A})\ddot{W}+l\vartheta^{A}\partial \ddot{W}+l^{2}(l^{2}m^{AB}+l^{2}{\hat{m}}^{AB}+\vartheta^{AB}){\ddot{V}}^{B}-\\ -l^{2}N^{AB}V^{B}+l^{2}K^{A}W+l^{4}K^{AB}V^{B}+l^{2}C^{A}\dot{W}+l^{4}C^{AB}{\dot{V}}^{B}+\underline{l^{3}{\tilde{\vartheta }}^{AB}\partial {\ddot{V}}^{B}}+\\ +\underline{l^{2}\partial \partial ({\bar{D}}^{A}\partial \partial W+l^{2}{\bar{D}}^{AB}\partial \partial V^{B})}+\underline{l^{2}[({\overset{⌣}{D}}^{AB}-4{\hat{D}}^{AB})-l^{2}{\bar{N}}^{AB}]\partial \partial V^{B}}-\\ -\underline{l^{2}\partial ({\bar{\vartheta }}^{A}\partial \ddot{W}+l^{2}{\bar{\vartheta }}^{AB}\partial {\ddot{V}}^{B}+l{\tilde{\vartheta }}^{AB}{\ddot{V}}^{B})}=l^{2}Q^{A}. \end{array}$$

By substituting Equation (18) into Equation (19) the averaged governing equations of the periodic beams can be written as:

(20) $$\begin{array}{l} \partial \partial [D\partial \partial W+D^{A}V^{A}+\tilde{B}\partial \partial \dot{W}+{\tilde{B}}^{A}{\dot{V}}^{A}+\underline{l^{2}\partial \partial ({\bar{D}}^{A}V^{A}+{\bar{B}}^{A}{\dot{V}}^{A})}]-\\ -\partial (N\partial W)+(\tilde{m}+\hat{m})\ddot{W}+l^{2}({\tilde{m}}^{A}+{\hat{m}}^{A}){\ddot{V}}^{A}-\partial (\vartheta \partial \ddot{W})-l\partial (\vartheta^{A}{\ddot{V}}^{A})+\\ +KW+l^{2}K^{A}V^{A}+C\dot{W}+l^{2}C^{A}{\dot{V}}^{A}-\underline{l^{2}\partial ({\bar{N}}^{A}\partial V^{A}+{\bar{\vartheta }}^{A}\partial {\ddot{V}}^{A})}=Q,\\ D^{A}\partial \partial W+D^{AB}V^{B}+{\tilde{B}}^{A}\partial \partial \dot{W}+{\tilde{B}}^{AB}{\dot{V}}^{B}+\underline{l^{2}{\overset{⌣}{B}}^{AB}\partial \partial {\dot{V}}^{B}}+\\ +l^{2}({\tilde{m}}^{A}+{\hat{m}}^{A})\ddot{W}+l\vartheta^{A}\partial \ddot{W}+l^{2}(l^{2}m^{AB}+l^{2}{\hat{m}}^{AB}+\vartheta^{AB}){\ddot{V}}^{B}-\\ -l^{2}N^{AB}V^{B}+l^{2}K^{A}W+l^{4}K^{AB}V^{B}+l^{2}C^{A}\dot{W}+l^{4}C^{AB}{\dot{V}}^{B}+\underline{l^{3}{\tilde{\vartheta }}^{AB}\partial {\ddot{V}}^{B}}+\\ +\underline{l^{2}\partial \partial ({\bar{D}}^{A}\partial \partial W+l^{2}{\bar{D}}^{AB}\partial \partial V^{B})}+\underline{l^{2}[2({\overset{⌣}{D}}^{AB}-2{\hat{D}}^{AB})-l^{2}{\bar{N}}^{AB}]\partial \partial V^{B}}-\\ -\underline{l^{2}\partial ({\bar{\vartheta }}^{A}\partial \ddot{W}+l^{2}{\bar{\vartheta }}^{AB}\partial {\ddot{V}}^{B}+l{\tilde{\vartheta }}^{AB}{\ddot{V}}^{B})}=l^{2}Q^{A}. \end{array}$$

Equation (20) stands the system of the governing equations of the general tolerance model of slender visco-elastic periodic beams on a periodic foundation with damping. They are the system for the macrodeflection W and the fluctuation amplitudes V^A^, A = 1, …, P, and they have a physical sense only if all these unknowns are weakly-slowly-varying functions in x. For every basic unknown: the macrodeflection W and the fluctuation amplitudes V^A^, A = 1, …, P, two boundary conditions should be formulated at both ends of the beam (four conditions together for every unknown). Characteristic features of Equation (20), similarly to Equations (18)–(19), are that they have constant coefficients and describe the effect of the microstructure size on the overall dynamic and stability behavior of the beams under consideration by terms involving the microstructure parameter l. Moreover, the governing equations of the general tolerance model include additional terms, which are underlined.

### 3.2. The (Standard) Tolerance Model Equations

By using the residual orthogonality condition Equation (16) with the modelling assumptions Equations (12)–(14) and the concept of the slowly-varying function, while introducing denotations of averaged coefficients similar to Equation (17):

(21) $$\begin{array}{lll} D\equiv <d>, & D^{A}\equiv <d\partial \partial g^{A}>, & D^{AB}\equiv <d\partial \partial g^{A}\partial \partial g^{B}>,\\ \tilde{B}\equiv <b>, & {\tilde{B}}^{A}\equiv <b\partial \partial g^{A}>, & {\tilde{B}}^{AB}\equiv <b\partial \partial g^{A}\partial \partial g^{B}>,\\ \tilde{m}\equiv <\mu >, & {\tilde{m}}^{A}\equiv l^{-2}<\mu g^{A}>, & {\tilde{m}}^{AB}\equiv l^{-4}<\mu g^{A}g^{B}>,\\ \vartheta \equiv <j>, & \vartheta^{A}\equiv l^{-1}<j\partial g^{A}>, & \vartheta^{AB}\equiv l^{-2}<j\partial g^{A}\partial g^{B}>,\\ C\equiv <c>, & C^{A}\equiv l^{-2}<cg^{A}>, & C^{AB}\equiv l^{-4}<cg^{A}g^{B}>,\\ K\equiv <k>, & K^{A}\equiv l^{-2}<kg^{A}>, & K^{AB}\equiv l^{-4}<kg^{A}g^{B}>,\\ \hat{m}\equiv <\hat{\mu }>, & {\hat{m}}^{A}\equiv l^{-2}<\hat{\mu }g^{A}>, & {\hat{m}}^{AB}\equiv l^{-4}<\hat{\mu }g^{A}g^{B}>,\\ N\equiv <n>, & N^{AB}\equiv l^{-2}<n\partial \partial g^{A}g^{B}>, & {\overset{⌣}{N}}^{AB}\equiv -l^{-2}<n\partial g^{A}\partial g^{B}>=-N^{AB},\\ Q\equiv <q>, & Q^{A}\equiv l^{-2}<qg^{A}>; & \end{array}$$

the following equations for unknowns *W*(⋅,*t*) and *V^A^*(⋅,*t*) are derived:

- The standard tolerance constitutive equations.
  (22) $$\begin{array}{l} M=D\partial \partial W+D^{A}V^{A}+\tilde{B}\partial \partial \dot{W}+{\tilde{B}}^{A}{\dot{V}}^{A},\\ M^{A}=D^{A}\partial \partial W+D^{AB}V^{B}+{\tilde{B}}^{A}\partial \partial \dot{W}+{\tilde{B}}^{AB}{\dot{V}}^{B}; \end{array}$$
- The standard tolerance equations of motion.
  (23) $$\begin{array}{l} \partial \partial M-\partial (N\partial W)+(\tilde{m}+\hat{m})\ddot{W}+l^{2}({\tilde{m}}^{A}+{\hat{m}}^{A}){\ddot{V}}^{A}-\partial (\vartheta \partial \ddot{W})-l\partial (\vartheta^{A}{\ddot{V}}^{A})+\\ +KW+l^{2}K^{A}V^{A}+C\dot{W}+l^{2}C^{A}{\dot{V}}^{A}=Q,\\ M^{A}+l^{2}({\tilde{m}}^{A}+{\hat{m}}^{A})\ddot{W}+l\vartheta^{A}\partial \ddot{W}+l^{2}(l^{2}m^{AB}+l^{2}{\hat{m}}^{AB}+\vartheta^{AB}){\ddot{V}}^{B}-\\ -l^{2}N^{AB}V^{B}+l^{2}K^{A}W+l^{4}K^{AB}V^{B}+l^{2}C^{A}\dot{W}+l^{4}C^{AB}{\dot{V}}^{B}=l^{2}Q^{A}. \end{array}$$

Equation (22) can be substituted into Equation (23); this leads to the averaged governing equations of the periodic beams in the following form:

(24) $$\begin{array}{l} \partial \partial [D\partial \partial W+D^{A}V^{A}+\tilde{B}\partial \partial \dot{W}+{\tilde{B}}^{A}{\dot{V}}^{A}]-\\ -\partial (N\partial W)+(\tilde{m}+\hat{m})\ddot{W}+l^{2}({\tilde{m}}^{A}+{\hat{m}}^{A}){\ddot{V}}^{A}-\partial (\vartheta \partial \ddot{W})-l\partial (\vartheta^{A}{\ddot{V}}^{A})+\\ +KW+l^{2}K^{A}V^{A}+C\dot{W}+l^{2}C^{A}{\dot{V}}^{A}=Q,\\ D^{A}\partial \partial W+D^{AB}V^{B}+{\tilde{B}}^{A}\partial \partial \dot{W}+{\tilde{B}}^{AB}{\dot{V}}^{B}+\\ +l^{2}({\tilde{m}}^{A}+{\hat{m}}^{A})\ddot{W}+l\vartheta^{A}\partial \ddot{W}+l^{2}(l^{2}m^{AB}+l^{2}{\hat{m}}^{AB}+\vartheta^{AB}){\ddot{V}}^{B}-\\ -l^{2}N^{AB}V^{B}+l^{2}K^{A}W+l^{4}K^{AB}V^{B}+l^{2}C^{A}\dot{W}+l^{4}C^{AB}{\dot{V}}^{B}=l^{2}Q^{A}. \end{array}$$

Equation (24) stands the governing equations of the standard tolerance model of slender visco-elastic periodic beams on a periodic foundation with damping. Similarly to Equation (20), they are the system for the macrodeflection W and the fluctuation amplitudes *V^A^*, *A* = 1 ,…, P. Two boundary conditions at both ends of the beam should be formulated only for the macrodeflection W (four conditions together) but not for the fluctuation amplitudes *V^A^*, *A* = 1, …, P. Characteristic features of these equations are that they: have constant coefficients; describe the effect of the microstructure size on the overall dynamic and stability behavior of the beams under consideration by terms involving the microstructure parameter l; have a physical sense only if all the basic unknowns *W* and *V^A^* are slowly-varying functions in x.

### 3.3. The Asymptotic Model Equations

In order to evaluate the above theoretical results (models) obtained in the framework of the tolerance modelling technique, the approximate model will be presented, the governing equations of which neglect the effect of the microstructure size. These equations can be derived applying the proper asymptotic modelling procedure (cf. [2,3] or directly from Equations (22)–(23)) after vanishing terms with the microstructure parameter *l*. Using some of the denotations in Equation (21) these equations can be written in the form:

- The asymptotic constitutive equations.
  (25) $$\begin{array}{l} M=D\partial \partial W+D^{A}V^{A}+\tilde{B}\partial \partial \dot{W}+{\tilde{B}}^{A}{\dot{V}}^{A},\\ M^{A}=D^{A}\partial \partial W+D^{AB}V^{B}+{\tilde{B}}^{A}\partial \partial \dot{W}+{\tilde{B}}^{AB}{\dot{V}}^{B}; \end{array}$$
- The asymptotic equations of motion.
  (26) $$\begin{array}{l} \partial \partial M-\partial (N\partial W)+(\tilde{m}+\hat{m})\ddot{W}-\partial (\vartheta \partial \ddot{W})+KW+C\dot{W}=Q,\\ M^{A}=0. \end{array}$$

After combining Equation (25) with Equation (26), the averaged governing equations of the periodic beams take the following form:

(27) $$\begin{array}{l} \partial \partial [D\partial \partial W+D^{A}V^{A}+\tilde{B}\partial \partial \dot{W}+{\tilde{B}}^{A}{\dot{V}}^{A}]-\partial (N\partial W)+\\ +(\tilde{m}+\hat{m})\ddot{W}-\partial (\vartheta \partial \ddot{W})+KW+C\dot{W}=Q,\\ D^{A}\partial \partial W+D^{AB}V^{B}+{\tilde{B}}^{A}\partial \partial \dot{W}+{\tilde{B}}^{AB}{\dot{V}}^{B}=0. \end{array}$$

Equation (27), similarly to Equations (25)–(26), represents the asymptotic model of slender visco-elastic periodic plates on a periodic foundation with damping, which neglects the effect of the microstructure size on the overall behavior of the beams. Two boundary conditions at both ends of the beam should be formulated only for the macrodeflection *W* (four conditions together). The above equations have constant coefficients, similarly to Equation (20) or (24) for the tolerance models, in the contrast to Equations (8)–(9) or (10) with non-continuous, highly oscillating, periodic, functional coefficients.

## 4. Results—A Simple Theoretical Example

### 4.1. Introduction

**Figure 2:**
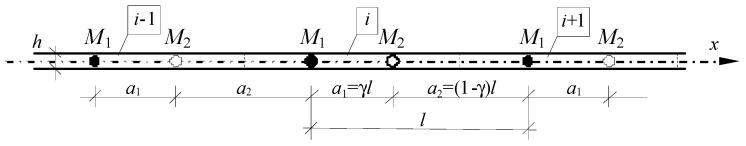
Fragment of periodic beam with a periodically distributed system of two concentrated masses, *M*_1_ and *M*_2_.

As an example, a homogeneous elastic unbounded beam without the effects of the rotational inertia, the axis force and the foundation is considered. Its periodicity is caused by the periodic distribution of the system of two concentrated masses *M*_1_ and *M*_2_ (Figure 2). 

**Figure 3:**
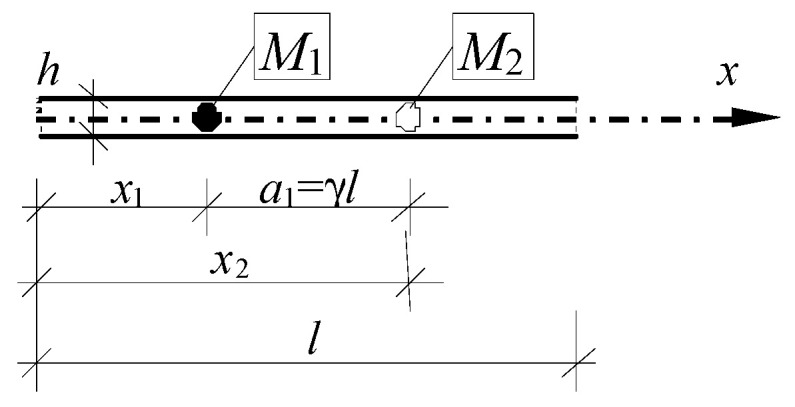
The periodicity cell of the considered beam.

Let us denote the distance between mass *M*_1_ and *M*_2_ by *a*_1_ and between *M*_2_ and *M*_1_ as *a*_2_ (hence *a*_2_ = *l* − *a*_1_); cf. Figure 3. 

Young’s modulus *E*, mass density μ, height *h* and width *b* of the beam are assumed to be constant. Load *q* is neglected. In considerations only one fluctuation shape function *g* = *g*^1^, *A* = *P* = 1, is assumed in the form:

(28) $$g(x)=l^{2}[\sin(2\pi x/l)+c],$$

where the constant *c* is calculated from the condition <μ*g*> = 0. The form of the fluctuation shape function *g* (Equation (28)) is related to the form of the periodicity cell shown in Figure 3. Denote also *V* ≡ *V*^1^.

Hence, the averaged coefficients (17) other than zero can be written as:

(29) $$\begin{array}{llll} d=\frac{1}{12}Ebh^{3}, & & & \\ D\equiv d, & D^{11}\equiv d<\partial \partial g\partial \partial g>, & \tilde{m}\equiv <\mu >, & {\tilde{m}}^{11}\equiv l^{-4}<\mu gg>,\\ {\bar{D}}^{1}\equiv l^{-2}d<g>, & {\bar{D}}^{11}\equiv l^{-4}d<gg>, & {\hat{D}}^{11}\equiv l^{-2}d<\partial g\partial g>, & {\overset{⌣}{D}}^{11}\equiv l^{-2}d<\partial \partial gg>. \end{array}$$

### 4.2. Example—Frequencies of a Travelling Wave

The aim of the example is to obtain formulas of frequencies of a travelling wave for the unbounded beam with the periodic distribution of the system of two concentrated masses *M*_1_ and *M*_2_ introduced above. 

#### 4.2.1. The General Tolerance Model

Using the averaged coefficients Equation (29) the governing equations of the general tolerance model Equation (20) for the considered beam can be written as:

(30) $$\begin{array}{l} D\partial \partial \partial \partial W+\underline{l^{2}\partial \partial \partial \partial {\bar{D}}^{1}V}+\tilde{m}\ddot{W}=0,\\ D^{11}V+l^{4}m^{11}\ddot{V}+\underline{l^{2}{\bar{D}}^{1}\partial \partial \partial \partial W+l^{4}{\bar{D}}^{11}\partial \partial \partial \partial V}+\underline{2l^{2}({\overset{⌣}{D}}^{11}-2{\hat{D}}^{11})\partial \partial V}=0. \end{array}$$

Introduce the wave number *k* (e.g., *k* = 2π/*L*). Solutions to Equation (30) will be assumed as:

*W*(*x*,*t*) = *A_W_*exp*i*(*kx* − ωt), *V*(*x*,*t*) = *A_V_*exp*i*(*kx* − ωt), (31)

with *A_W_*, *A_V_* as amplitudes, ω as a frequency. By substituting Equation (31) into Equation (30) the following system of algebraic equations is obtained:

(32) $$\begin{array}{l} (Dk^{4}-\tilde{m}\omega^{2})A_{W}+\underline{l^{2}{\bar{D}}^{1}k^{4}}A_{V}=0,\\ \underline{l^{2}{\bar{D}}^{1}k^{4}}A_{W}+[D^{11}+\underline{l^{4}{\bar{D}}^{11}k^{4}}-\underline{2l^{2}({\overset{⌣}{D}}^{11}-2{\hat{D}}^{11})k^{2}}-l^{4}m^{11}\omega^{2}]A_{V}=0. \end{array}$$

While comparing the main determinant of the above system of equations to zero, the characteristic equation can be written as:

(33) $$\begin{array}{l} l^{4}\tilde{m}m^{11}\omega^{4}-\{\tilde{m}[D^{11}+\underline{l^{4}{\bar{D}}^{11}k^{4}}-\underline{2l^{2}({\overset{⌣}{D}}^{11}-2{\hat{D}}^{11})k^{2}}]+l^{4}m^{11}Dk^{4}\}\omega^{2}+\\ +[D^{11}+\underline{l^{4}{\bar{D}}^{11}k^{4}}-\underline{2l^{2}({\overset{⌣}{D}}^{11}-2{\hat{D}}^{11})k^{2}}]Dk^{4}-\underline{{\left(l^{2}{\bar{D}}^{1}k^{4}\right)}^{2}}=0, \end{array}$$

from which the following formulas of the travelling wave frequencies in the framework of the general tolerance model can be obtained as

(34) $$\omega_{-,+}=\sqrt{\begin{array}{l} \frac{\tilde{m}[D^{11}+l^{4}k^{4}{\bar{D}}^{11}-2l^{2}k^{2}({\overset{⌣}{D}}^{11}-2{\hat{D}}^{11})]+l^{4}k^{4}m^{11}D}{2l^{4}\tilde{m}m^{11}}\mp \\ \mp \frac{\sqrt{{\left\{l^{4}k^{4}m^{11}D-\tilde{m}[D^{11}-2l^{2}k^{2}({\overset{⌣}{D}}^{11}-2{\hat{D}}^{11})+l^{4}k^{4}{\bar{D}}^{11}]\right\}}^{2}+{\left(2l^{4}k^{4}{\bar{D}}^{1}\right)}^{2}\tilde{m}m^{11}}}{2l^{4}\tilde{m}m^{11}} \end{array}},$$

where ω_−_ is the fundamental lower frequency related to the averaged macrostructure of the beam with the periodic distribution of the system of two concentrated masses, but ω_+_ is the higher frequency related to the microstructure of the beam.

#### 4.2.2. The Standard Tolerance Model

By applying the averaged coefficients Equation (29) the governing equations of the standard tolerance model Equation (24) for the considered beam can be written as:

(35) $$\begin{array}{l} D\partial \partial \partial \partial W+\tilde{m}\ddot{W}=0,\\ D^{11}V+l^{4}m^{11}\ddot{V}=0. \end{array}$$

It should be noted that, in contrast to the general tolerance model, two independent differential equations of the beam under consideration are obtained. The first of them describes vibrations of beam for a “macro” scale, and the second equation determines “microvibrations” related to a periodic system of two concentrated masses. By substituting solutions of Equation (31) into Equation (35) the uncoupled system of algebraic equations can be written in the form:

(36) $$\begin{array}{l} (Dk^{4}-\tilde{m}\omega^{2})A_{W}=0,\\ (D^{11}-l^{4}m^{11}\omega^{2})A_{V}=0. \end{array}$$

The characteristic equation in this case takes the form:

(37) $$l^{4}\tilde{m}m^{11}\omega^{4}-(\tilde{m}D^{11}+l^{4}m^{11}Dk^{4})\omega^{2}+D^{11}Dk^{4}=0,$$

The formulas of the travelling wave frequencies in the framework of the standard tolerance model have the form:

(38) $${\tilde{\omega }}_{-}=\sqrt{\frac{Dk^{4}}{\tilde{m}}},\;{\tilde{\omega }}_{+}=\sqrt{\frac{D^{11}}{l^{4}m^{11}}},$$

where ${\tilde{\omega }}_{-}$ is the fundamental lower frequency related to the averaged macrostructure of the beam with the periodic distribution of the system of two concentrated masses, but ${\tilde{\omega }}_{+}$ is the higher frequency related to the microstructure of the beam.

#### 4.2.3. The Asymptotic Model

Using the averaged coefficients Equation (29) the governing equations of the asymptotic model Equation (27) for the considered beam can be written as:

(39) $$D\partial \partial \partial \partial W+\tilde{m}\ddot{W}=0,\;D^{11}V=0.$$

It should be noted that, in contrast to both the tolerance models—general and standard, only one differential equation of the beam under consideration is obtained. This Equation (39)_1_ describing vibrations of beam for a “macro” scale and the effect of the microstructure of the beam, in the form of the higher order vibrations, is neglected. By substituting Solutions (31) to Equation (39)_1_ the algebraic equation is obtained:

(40) $$(Dk^{4}-\tilde{m}\omega^{2})A_{W}=0,$$

which leads to the characteristic equation in the form:

(41) $$\tilde{m}\omega^{2}-Dk^{4}=0,$$

but the formula of the travelling wave frequency in the framework of the asymptotic model is:

(42) $$\omega_{0}=\sqrt{\frac{Dk^{4}}{\tilde{m}}},$$

where ω_0_ is the fundamental lower frequency related to the averaged macrostructure of the beam with the periodic distribution of the system of two concentrated masses The effect of the microstructure of the beam in from of the higher frequency is omitted.

### 4.3. Comments on the Example

By analyzing results obtained in this simple example it can be observed that both the tolerance models—general and standard, allow one to investigate the effect of the microstructure in dynamical problems, which occurs in the possibility of considering higher order vibrations related to the microstructure. In addition, it seems that the general tolerance model better describes the lower order fundamental vibrations, taking into account the effect of microstructure. Compared with both the tolerance models, the asymptotic model only allows one to study lower order fundamental vibrations.

## 5. Remarks

By summing up the above theoretical considerations, some remarks can be formulated.

- The tolerance modelling method allows one to derive averaged equations of models of slender visco-elastic beams on a periodic damping foundation with constant coefficients, which replace the classic equations with non-continuous, periodic coefficients.
- Using various definitions of concepts of the weakly-slowly-varying function and the slowly-varying function. two different tolerance models can be derived in the framework of the tolerance approach—the general tolerance model and the standard tolerance model.
- The governing equations of both the tolerance models involve terms which describe the effect of the microstructure size on overall dynamic and stability behavior of these beams; hence both models allow one to analyze dynamic and stability problems of the beams under consideration on the macro-and the micro-level.
- The form of the model equations depends on the class of slowly-varying, basic, unknown functions. The weakly-slowly-varying functions lead to the general tolerance model equations, which involve additional terms depending on the microstructure parameter; but the slowly-varying functions lead to the standard tolerance model equations.
- In contrast to the governing equations of the standard tolerance model for slender visco-elastic beams on a periodic damping foundation, the equations of the general tolerance model for these beams include additional terms describing the effect of the microstructure size also in stationary problems.
- In contrast, the asymptotic model allows one to analyze dynamic and stability problems of the beams under consideration only at the macro-level, without the effect of the microstructure.
- Based on the presented simple example, it can be observed that the proposed general tolerance model also allows one in such a simple case not only to analyze the effect of microstructure in the form of higher order vibrations (microvibrations), but also to consider and relate this effect with the fundamental lower order vibrations (macrovibrations).

Some applications of the general tolerance model equations of the slender visco-elastic beams on a periodic damping foundation will be presented in forthcoming papers. It also seems that the proposed tolerance models can be used in this or suitably adapted form in the analysis of problems related to nanostructures, e.g., nano-beams, but it is an open problem.
